# Editorial: Molecular Aspects of Mucopolysaccharidoses

**DOI:** 10.3389/fmolb.2022.874267

**Published:** 2022-02-28

**Authors:** Grzegorz Węgrzyn, Karolina Pierzynowska, Luigi Michele Pavone

**Affiliations:** ^1^ Department of Molecular Biology, Faculty of Biology, University of Gdansk, Gdansk, Poland; ^2^ Department of Molecular Medicine and Medical Biotechnology, School of Medicine, University of Naples Federico II, Naples, Italy

**Keywords:** mucopolysaccharidoses, glycosaminoglycans, lysosomal storage diseases, genetic variations, genotype-phenotype correlation

Mucopolysaccharidoses (MPS) are a group of 13 diseases (see Table 1 for details) belonging to lysosomal storage disorders (LSD) that occur with cumulative frequencies of all their types of about 1 per 40,000—50,000 live births (Çelik et al., 2021). Out of these 13 types of MPS, 11 are known since many years, while 2 types were discovered recently, MPS X (Verheyen et al., 2022) and MPS-plus-syndrome (MPSPS) (Vasilev et al., 2020). All MPS diseases are caused by mutations in genes coding for enzymes involved in metabolism (usually catabolism) of glycosaminoglycans (GAGs) (formerly called mucopolysaccharides). Their impaired hydrolysis or other stage(s) of metabolism leads to continuous accumulation and storage of these compounds in cells of patients which results in a damage of the affected tissues, including the heart, respiratory system, bones, joints, and central nervous system (Fecarotta et al., 2020). MPS are usually fatal diseases (especially neuronopathic forms of MPS), with average expected life span less than 2 decades (or significantly shorter in some cases). MPS are monogenic diseases, which are often considered model genetic diseases in studies on mechanisms of genetic disorders and development of novel therapeutic strategies (Tomatsu et al., 2018).

**TABLE 1:** Types of mucopolysaccharidoses (MPS).

MPS type^a^	Primary stored GAG(s)	Mutated gene	Deficient enzyme/protein
MPS I	Heparan sulfate, dermatan sulfate	*IDUA*	α-L-iduronidase
MPS II	Heparan sulfate, dermatan sulfate	*IDS*	Iduronate-2-sulfatase
MPS IIIA	Heparan sulfate	*SGSH*	N-sulfoglucosamine sulfhydrolase
MPS IIIB	Heparan sulfate	*NAGLU*	α-N-acetylglucosaminidase
MPS IIIC	Heparan sulfate	*HGSNAT*	Acetyl-CoA:α-glucosaminide acetyltransferase
MPS IIID	Heparan sulfate	*GNS*	N-acetylglucosamine-6-sulfatase
MPS IVA	Keratan sulfate, chondroitin sulfate	*GALNS*	N-acetylgalactosamine-6-sulfatase
MPS IVB	Keratan sulfate, chondroitin sulfate	*GLB1*	β-galactosidase-1
MPS VI	Dermatan sulfate	*ARSB*	N-acetylgalactosamine 4-sulfatase
MPS VII	Heparan sulfate, dermatan sulfate, chondroitin sulfate	*GUSB*	β-glucuronidase
MPS IX	Hyaluronic acid	*HYAL1*	Hyaluronidase-1
MPS X	Heparan sulfate, dermatan sulfate, keratan sulfate, chondroitin sulfate	*ARSK*	Glucuronate 2-sulfatase
MPSPS	Heparan sulfate, dermatan sulfate	*VPS33A*	Vacuolar protein sorting-associated protein 33A

a Summary on mucopolysaccharidoses I-IX was presented by Tomatsu et al. (2018), MPS X has been discovered recently by Verheyen et al. (2022), and MPS-plus-syndrome (MPSPS) was characterized recently by Vasilev et al. (2020) (note that an MPS plus-like syndrome has been described in patients with mutations in the *VPS16* gene, coding for vacuolar protein sorting protein 16; Yıldız et al., 2021).

Although MPS are monogenic diseases, recent studies demonstrated that their pathomechanisms are significantly more complex than primary effects of GAG storage on lysosomal functions. It appeared that expression of hundreds of genes is changed in MPS cells (Gaffke et al., 2020). This causes further changes in proteome (De Pasquale et al., 2020a) and metabolome (De Pasquale et al., 2020b), and resultant disturbances of structures and functions of cellular organelles and many cellular processes (Gaffke et al., 2021), finally leading to dysfunctions of tissues, organs, and the whole body. Nevertheless, our knowledge on the molecular processes occurring in MPS cells, and disease-specific changes relative to normal cells, is still limited. On the other hand, understanding molecular pathomechanisms of the disease, especially molecular aspects of various interactions between biologically active macromolecules, is crucial for both gaining basic knowledge on regulation of various biological processes and developing new therapeutical strategies for genetic diseases which are particularly difficult to cure. In fact, previous studies on MPS allowed to achieve spectacular progress in understanding principles of genetic disorders and to propose many sophisticated therapeutical options, including enzyme replacement therapy, substrate reduction therapy, modifications of gene therapies, and many others (McBride and Flanigan 2021). Interestingly, studies on MPS provided a basis for intriguing proposals concerning diseases from other groups, including unexpected aspects of COVID-19 (Pierzynowska et al., 2020; De Pasquale et al., 2021).

Therefore, a special issue of this journal has been devoted to present papers which can increase our knowledge on the molecular aspects of MPS. This should provide broader view on these diseases, indicate possible novel tools for their analyses, and have significantly broader meaning for bio-medical sciences.

Communication and discussion of all valuable data connected to rare diseases, like MPS, are crucial elements for the development of a more complete understanding of these disorders. Low number of patients causes limitations of sources of important data, making each solid result especially valuable. This problem has been discussed by Sampayo-Cordero et al. who presented the deleterious effects of excluding results of non-randomized studies from systematic reviews. The authors demonstrated that such a strategy of selection of data in reviews may result in a loss of a considerable amount of information in MPS and most probably in other rare diseases. Different strategies for combining data from randomized and non-randomized studies were discussed, and important recommendation for including results of both these types of studies in systematic reviews on MPS was proposed which should allow to both avoid a selection bias and increase the representativeness of the results.

Genotype-phenotype relationships are important issues when predicting severity of MPS disease in patients at the time of diagnosis, as proper prediction might be crucial to choose the most effective management which is still of low efficiency. Unfortunately, such relationships are often questionable, and the level of difficulty of extrapolation of our knowledge based on previously described cases and molecular data on biochemical effects of specific mutations is very high. *In silico* programs provide tools for analyses of mutations and their possible effects, however, their reliability is unclear, especially when missense variants are investigated. An interesting comparison of 33 such programs in studies on mutations occurring in the *IDUA* gene (causing MPS I) has been presented by Matte et al. They analyzed 586 missense mutations in this gene and found that the ClinPred algorithms gave the best sensitivity, specificity, accuracy, and kappa value for two criteria established by the authors. This can be an important suggestion for further analyses of mutations in MPS I and other types of this disease.

A very important review by Vuolo et al. is focused on reproductive parameters occurring in animal models of MPS and other LSD. The crucial conclusions proposed by the authors are that 1) many animal MPS models show alterations in the reproductive system, but 2) vast majority of studies were performed with the use of males only, therefore, 3) it is highly recommended to use also female animals in laboratory studies which should allow us to better understand the physiopathology of MPS.

Two articles by Voskoboeva et al. present epidemiology and genetics of MPS I (Voskoboeva et al.) and MPS VI (Voskoboeva et al.) in Russia and some neighbor countries. Since this is a large territory, such analyses are very valuable for understanding basic processes of the cause of the diseases, geographical distribution of patients, potential effects of the founder, and other mechanisms related to epidemiology of rare diseases. Moreover, the presented results indicated feasibility of the newborn screening procedures in the country and suggested optimal diagnostic tools in specific ethnic groups.

Finally, the study by Brusius-Facchin et al. indicated usefulness of the Next Generation Sequencing (NGS) in detection of carriers of MPS II. This disease is the only example of the X-linked disorders among all MPS types. Thus, proper identification of female carriers is especially important. Moreover, the use of NGS allowed to detect mosaicism in mothers of MPS II patients which was more accurate than classical DNA sequencing. This can be recognized as an important methodological improvement in assessment of the risk of MPS II.

In summary, the articles published in this special issue indicated important methodological optimizations for studies on molecular aspects of MPS and provided valuable epidemiological and genetic data on selected types of MPS. Thus, readers are encouraged to read details of these studies, and to follow the molecular aspects of MPS in their further investigations.
